# Supplementary material for: Community perceptions and experiences on caring for the premature babies in Arba Minch health and demographic surveillance site, southern Ethiopia: Interpretive Husserlian phenomenological study
Source: PLoS One. 2024 Jan 2;19(1):e0294155. doi: 10.1371/journal.pone.0294155 (PMC10760645; doi:10.1371/journal.pone.0294155)
Supplement: S2 Dataset — (DOCX) [file pone.0294155.s003.docx]

**Qualitative Data Transcription and Translation_ AM-HDSS, 2022**

One of the FGD participants said that pre-term baby means a baby delivered before nine month. The baby delivered in seven month is challenging as the families are in trouble and stressed as either the baby survive or not. The mother is faced a challenge if she give birth of pre-term baby and if God allowed such babies may grow. The difference between pre-term and low-birth weight baby is that, the low birth weight is only small or weak, but healthy. Nevertheless, pre-term baby is also small and unable to suck the breast, as it is not mature. The pre-term babies must putted in cotton until eight or nine month to give warm and avoid from cold. If God allowed such babies may grow. One of the FGD discussants (female) stated that pre-term babies are does not suck and it is challenging to feed them. Pre-term babies are very weak and small. The discussant stated that it is possible to recognize pre-term babies by observing. As pre-term babies are weak, as immature, her body is semis like paralyzed, unable to suck and breast-feed. It is difficult to know whether the pre-term babies grow or not and it depends. If God help and effective care given the babies grow. Unless, it does not survive.

The FGD participants states that the cause for pre-term delivery is carrying high loaded materials during pregnancy. If the women become pregnant, she must kept herself from such activity to get healthy child. Health professionals also counsel about this during pregnancy in the antenatal care. The cause for pre-term delivery is sin “Gome” or “lanche” or some is disappointed on some activities in the family member or relative (father or mother, etc.).

As one of the FGD discussant stated that the pre-term babies must kept in cotton or wollen (Netela) to warm the body and to avoid from bruising, as the body is very soft. It is difficult to breastfeeding the pre-term baby and the mother as well as other must support and try to breastfeed the baby for a period. The body if pre-term baby must washed continuously (two times per a day). Unless, the body my decay.

The FGD discussant stated that, if the mother have pre-term baby it limits her from social participation as a baby need close follow up and it is difficult to give pre-term babies for other individuals or care givers. As such, the neighbors and relatives may support her and the local peoples as well as kebeles leaders understand the situation and the give permission for the family until the baby strengthen him/herself. Growing pre-term baby is very challenging as psychosocially and economically affects the family. If pre-term baby born in the surrounding and the community nearby as well perceived that, this baby does not survive and always taking about that baby and this creates a great stress on family members.

One of the FGD discussant stated that my son wife also give birth of pre-term baby in seven month in the hospital. The baby admitted to NICU and the health care professionals give care for the baby putted in the incubator. Previously, most pre-term babies cannot survive as there is no that much advanced care and skilled/trained health professionals as we are living rural far too rich area. However, nowadays most pre-term babies survive as care provided by the health professionals and if God supported.

The discussants stated that the main challenge related with this that it difficult to rich the pregnant mother to hospital, as the road is not we structured ad we can access transportations. As, the pregnant mothers suffering more to rich to hospitals as there is no any hospital in our surrounding and the mother must go to Arba Minch General hospital via transport if it referred from local health center and if complications occur. We carry the pregnant mother via cahier or bed to rich the main road to take to Arba Minch hospital.

The health professionals in the health center give counseling the pregnant mother during antenatal care to avoid caring high loaded materials, to have balanced diet, and to keep herself from any situations that harm the mother as well as the fetus.

One of the FGD discussant stated that pre-term baby means a baby which born in seven or eight month of pregnancy. The baby may survive or not. The other member of the FGD, said pre-term baby means as my friend stated a baby which either born in seven or eight month or before completing nine month. The baby born in seven month can survive. However, if it born in eight month it cannot survive completely. The third male participant that get the chance to took forwarded that as my brother said pre-term baby is a baby born in seven or eight month or before nine month. Again, the other FGD discussant (male) stated we could differentiate pre-term baby from term babies as term babies are born healthy and strong. However, when we observed pre-term babies are born early or months are left to born and out of human personality as very weak, cannot suck mother breast (feed breast milk) and it may stay in incubator (NICU) for some period before come contact with the mother. The other energetic man (he is a local police or milisha) stated when a term and pre-term babies are putted in the incubator; the pre-term babies that born in seven month cannot show any range of motion from placed position as it is weak. But, the term baby can show movement (flex and extend the extremities) as month is complete, can cry to indicate the need of breast for feeding, the health professional’s continuously follow and observe for the existence and some may survive after those situation. By such situation, it survive and when months are completed, it start to breast feed. Therefore, we can differentiate the seven-month baby from the nine month in characteristics. Both term and pre-term babies are putted in the incubator and the seven-month baby can show any movement. As to me, we cannot differentiate low birth babies from pre-term baby rather than the health professionals. I differentiate my baby as pre-term as the father said it is very weak and the body is like blood as it is not mature and the body is transparent (has hole), as term babies are strong, but my baby does not completed nine month (two month is left) and the body is not connected (mature enough). We easily observe the bone of the baby, when after my baby was born, I have not believed the doctors information as he said the baby may survive. I overserved that the baby’s body as it is totally hole (transparent) and easily observed the hand of other personal putted behind. The skin is very soft.

Regarding the cause, one of the female FGD discussant said that carrying water jar or “Jarkine” or having heavy load can make it change the position of the baby as the pressure is not comfortable, it also result for cold prolapse and the baby may delivered while the cord is knotted or strangulated in the neck. I know this as situation as my neighbor informed from the health care institution as the placenta is on the head (in front of the baby) and she delivered after two-month stay in seven month. The other cause for pre-term delivery as one of the FGD discussant stated, if the mother malaria and cannot treatment soon, the baby may delivered pre-termly “shochetidi kees”. After get sick, if the mother delayed, they baby is harmed. One of the old man said I have also a child delivered in seven month, but now he grow and become adolescent. Initially, I challenged and I do not believed for the survival. Nevertheless, I do not the exact cause for pre-term delivery. The other participant (mother) stated the cause is carried heavy loaded material, pregnancy mother cannot allowed to grind coffee in grinder or mortar, must get vaccination during pregnancy. The government also not allowed pregnant women to do heavy activities, as she did that activities it may cause preterm delivery and it cause harm.

One of the FGD participant stated my wife delivered pre-term baby due to sickness rather than carrying heavy loaded material. She critically ill and she give birth at seven month of pregnancy. To grow pre-term babies, it need to give himself, consulting health professionals and traditional healers needed to get information how to manage such type of babies. One of the neighbor said why you tired yourself as this baby could not survive. Nevertheless, I strongly responded that wife severely ill but she cannot die as the life in hand of God. As such, this baby cannot die. I believed that if families (mother and father) must give himself or herself for child to grow, to keep or to give care. Unless, the baby cannot survive. When we say, “giving self,” mean that must assemble all the necessary supplies for the baby, and consult professionals. The other participant reported to grow pre-term babies, it needs close follow-up, giving him/herself, consult health professionals and traditional healers. Unless, pre-term babies cannot survive and grow. The other FGD participant stated, as my wife become pregnant and become ill until delivery at seven month. Even after she give birth, the health professionals assessed her and she has fever. After 6-day stay in the incubator, the health professionals planned to attach the baby with mother as skin-to-skin contact needed, but has fever. As such, the health professional informed me that this fever might transmit to the baby if we directly attached to the mother. So, as you are the father of the baby, you can also possible to give skin-to-skin contact for the baby. After that, I sited in litter chair, putted the baby in chest and wearing a coat to give warmth, and I stayed for 1-4 hours like Monkey. After getting my warm for the four-hour and the baby-started movement for searching breast to feed. As such, the professional’s took the baby to attached with mother. Nevertheless, the mother has no milk, b/c she is “Findata”. The health care providers expressed her breast to get milk and they trained to for the baby via syringe. B/c, the baby unable to suck to get the milk. They also tried to feed the expressed breast milk by “NGT” for 24 hour in three consecutive days. Consequently, the baby got strength, and started to suck and get milk from mother breast.

One of FGD discussant stated that pre-term baby needs critical follow up care until grow, and the mother who give birth of pre-term baby needs support. It also needs consultation from professionals on feeding the baby as well as the mother him, and practicing those activities are very important. My baby is stayed in one week in NICU; we are challenged to feed until started mother breast. As such, the HP feed expressed milk by syringe. It is needs care for the mother and additional artificial prepared milk. It needs hygiene for the mother and the baby. Therefore, we washed the baby twice a day. The other traditional healer old man said that mother’s faces the main challenge either during pregnancy, or during caring for the child. The husband is simply as the name husband rather than sharing the burden of the wife.

A mother of pre-term baby stated my baby was born at seven month and the birth weight is not much small, it is two point some KG. After birth, it admitted to NICU during the first day of life, as the baby is unable to suck. In the second day, the baby developed fever; I understand that they give fluid via syringe and other medications. In the four day, I expressed my breast and they gave by a tube by nose. After that, in the fifth day, blood comes on his nose and mouth as the baby not admitted to feeding, and the HP become disturbed. They cleaned the blood from the mouth and took sample for analysis. Initially, the baby body is while and weak while observed. Starting from the day that the blood comes in nose and mouth, he continuously cry and we all family members stressed and we raised a question that “Does this baby survive and grow?” and me and my husband also decided that this baby may die. Due to continuous cry, the health professional’s get out from the NICU, and they informed me to give warmth by putted between my breast and at night around 5 O’clock he sucked for a few moment. After two days of sucking breast and the bleeding stopped, we discharged from hospital to home. Then, the baby was stable six month. At the six month my faced a diarrhea. I kept the hygiene of the baby by washing his body and cloth, and I washed by breast before giving to him to suck. The other thing is that we also limited the number of persons who visit as informed from health care professionals. To give adequate amount of breast milk, I also kept my balanced diet. Caring pre-term baby is very challenging, but if the mother give, adequate care and follow up it grow by the help of “God”. I kept his personal hygiene by changed the cloth, washing breast before breast-feeding, and continuously I washed the body always at 10:00AM let it get to sleep. Even it is difficult to give care for pre-term babies, but I have not that much challenged as compared while my baby was at hospital.

One of the women who have pre-term baby stated that, now my baby is aged 2 year and five month, we faced some challenges related to the social life, as it is difficult to put the baby with the other personal’s as it cough and vomiting the ingested material. As such, we alternatively (with my husband) go and participant in social activities like “lekiso”, and other related social activities. Even if, my neighbor call let to go to funeral ceremony, I told to them that there is no any person to keep my baby until my husband comes. So, informed them to go. As such, having pre-term baby limit social participation. The other FGD participant related with challenge or influence from th community due to having pre-term baby stated that as pre-term baby needs care, the mother must be raised in home and care (providing good nutritional foods) should be given for him, and limiting the workload on the mother is necessary. Rather than those stated, there is no challenge as compared with having a mother with term baby.

The other energetic man reported that there is great difference in having pre-term and term babies. In my case, I come back to my home after 1 month and 7 days from hospital. I am in stress that, there are pregnant women’s in my neighbor while my baby was born and those women give birth after two days, while I come back to home, but those babies crawled, and walked in leg early than my baby. Due that case, I stressed and I prayed to “God” to help me that my baby was born early, but he cannot crawl as that of babies delivered late. Then, my again trained to crawl and to let to walk by hold materials near to him like a “plastic chair, table and others. Consequently, it stated to walk after a month back of those babies delivered late. However, now he equally walk and play with those babies even if there was a difference. Again, there also a challenge that it limits social life as baby is aged 2 year and 2 month now, but my wife is not actively engaged in social participations such as “Idir” as the baby only knows and adapted her and me only and he continuously cry, unstable, and he is a difficult child. In some situations, she kept the baby on my father (grandfather) for child, to fetch water if me are not around. In conducing household activities, I have banana plantation and when I was in hospital I completely attention for the baby and I do not who selling my banana either blockers or my family. So, there is a great gap was created in life as the professional ordered different medications for the baby and informed me to buy and I bought on request.

In our case related to social life, the community understands that it is not mandatory until six month whether the baby delivered term or pre-term. If the attendances called in “Idir” “furinal ceremony”, and other activities, there neighbor informed that she is “mechat”.

A women who worked as traditional birth attendant in 1989 E.C reported that I let too got to home of labored mother when they called me to come. I have a hand glove and I assessed the mother whether it possible for to deliver at home, and if it is ok I assisted the mother the deliver at home. Unless, I took the mother to health center by supporting him. I other situation, if the delivered at home by the help of me and if the placenta was not delivered again I took the mother to hospital including the delivered baby. Before, delivering the mother I boiling the bleed and a cold tie at least 30 minute and I cut the cold by that bleed and tie with the cold tie. Then, I registering the sex and the date birth of the baby and kept it with me and I reported to health post. One thing that I faced is that a women was gave birth at home without getting help from other and I arrived late after she gave birth, and the placenta was retained, and then the women way took to hospital. Nevertheless, the women died when the health professional is on way and assessing to help her. In addition, I continuously monitor the growth of child and if they become weak, I provide a food or “plampinet” for those wasted children after discussion with professionals in the health center, I informed for all to get vaccine based on the schedule or any vaccination campaign arranged. Rather that, I have not faced a pre-term baby in my career life.

One of the FGD discussant stated that while caring for pre-term babies, we got different counseling from health professional’s on feeding the baby, hygiene, caring the mother. If the mother breast has no milk, we also gave boiled cow milk by pacifier. The other discussant stated the pre-term babies could not survive without the health professionals in nearby as they critically follow, give counseling for us regarding feeding, examine for different diseases and provide medications.

Regarding the causes for pre-term baby, the discussants stated our father’s a said conflict between couples or sin or “Goome” my cause’s pre-term delivery. Nevertheless, now this is also true as peace is very important to live together in harmony and the babies delivered in good health and grow well. Unless, if there is any conflict in home, it disturbs the life of family members.

The other participants raised the situation how he informed about the wife condition. I am farmer and also working with my friends in weighing a banana and the individuals called to me via cellphone that oh…… your wife was going to hospital after having labor and when until I arrive at home they took my husband to Abriham clinic and she was admitted after assessment. After I arrived to the clinic and I am very confused reading what assessment done, what medications administered. I have only 1600 ETB as suddenly go to clinical after getting the information. However, after sum upped the ordered medications and the others service within an hour that I arrived to her, it costed 4400ETB. After that, I come back to home to get money from others I called to Bajaj as the time was “10:00PM”. Then after 1600ETB from others, I returned her clinic to home before getting to hospital.

One of the FGD discussant raised a compliant on local health workers, “health care provider in health post” on follow up pre-term babies. After discharge from hospital as my brother is Doctor and he wrote all the information about my baby and informed me to give this written paper for HEWs in which the baby need close follow up from them. Nevertheless, they does not gave any support for my baby as my baby is unstable, ear infection, and continuously ill, even they does not asked me about the situation. Due that case, I consulted traditional healer, he ordered traditional herbal medicine and informed me to re-visit again after two days, and he provided the medicine again. Then, my baby become stable and the health condition improved without going to health care institution and without consulting any health care provider. The herbal given for the baby was for “evil eye”.

One of the FGD participant (women) said that pre-term means if baby delivered live in seven month and if baby delivered at seven month and it is not live, we call it as “Bosha”. If a baby delivered below seven month or above seven month, but below nine month, it cannot survive. Pre-term babies are different from low-birth babies, as pre-term babies are very weak, the health professional delivered as they skillful, and it needs the warmth of mother “kangaroo mother care”. In addition, to what she said there is blood which transferred to the fetus during seven month and the mother must be healthy to get healthy baby. If the mother is not healthy, the baby that delivered from that mother is not healthy. Therefore, there is a difference. My baby delivered at seven month, and during seven months of pregnancy, hypertension diagnosed, and before I had also anemia and this changed to hypertension. Due to that case, my fetus unable to get health blood. After that, my baby delivered at seven month and it looks like “a rat that drown to water”. If the baby delivered at nine month the weight is 4 k.g, but mine is 1.8 k.g female baby and the Doctor gave a baby and informed this may or may not survive. When they putted in incubator, the baby unstable and the assessed the health condition, the baby was well, but very weak. The Doctor delivered me as the blood pressure is very high and they decided that the health of mother must took the first priority and they decided for the termination of pregnancy. However, they challenged that the baby not become stable while they putted in “incubator”. After that, we are discharged from hospital and they informed me that the baby is healthy and put in your chest and swaddle with cloth to warmth (kangaroo mother care), and if the observe any change in health condition please re-visit again the hospital. After that, weight increased from time to time as the baby get warmth from my body, and grow it until five month. On other hand, if the mother is healthy the baby delivered from her approximately 2 or 3 k.g, but it putted in “incubator” until nine month. The “incubator” gives warmth as the baby is in the mother “womb”. Therefore, the mother must wait in the hospital until the baby filled nine month. After the baby completed nine month, come out from “incubator”, and grow like that of baby delivered in nine month.

The other FGD discussant stated if the mother give birth before seven month it called “Bosha” or “abortion. If the give birth in seven month we called that the delivered pre-term baby. If the baby delivered before seven month it cannot survive, but if delivered in seven month it can survive. I observed and I told to the mother that why brought this baby to home, do you think that this baby grow?. The mother of the women who gave birth of pre-term baby also refused to took the baby while they discharged from the hospital. We also informed to throw the baby in toilet. Likewise, the baby delivered in seven month, survived unit now and it grow.

The other FGD participant told a heart broking history that, her daughter give birth at seven month due to hypertension. Her life was path away due to the hypertension after one week of delivery. I provided the care and I grow the baby until now. However, it very challenged that the baby facing diarrhea, vomiting, and tonsillitis as it is weak until now, because he cannot get the breast milk. I faced such situation as the mother died and I challenged as that of the mother give birth or pre-term baby. The father of the women who loosed her life also stated that my wife and I grow this baby as we bought different formula milk, other necessary things for the baby. Nevertheless, the walking of the baby is not correct as the age was 4 years now. Most people informed as not give any food from home as it is prepared foods in the mini market, and we bought those commodities. The baby looks like a little “chicken”, we challenged and we took vomitus, and diarrhea. Until now, if we took the baby to health institution, they told for us the baby has malnutrition, and they asked that “Does the mother is not available for the baby?”, and “Does the mother not feed the baby”. After that, we told to them that the mother died, and the health professional become anger. The baby can walk, play with others child, but he is very weak and his anatomical posture is not correct because he does get breast milk. Until now, we are in challenge due to this baby. As such, the seven month baby can survive, but with a great challenge. Therefore, the seven-month baby if get the mother milk, and may or may not survive if the baby cannot breast milk, but the six and the eighth month baby cannot survive.

Regarding causes for pre-term birth, one of the FGD participant (male) reported, our community perceived it as trend or culture, the cause for pre-term birth is carrying high loaded material, or dough bucket resulted and a large pot. However, this is the community perception and we do not the exact cause for pre-term delivery. The pre-term may occur during six, seven or even in nine month. My sister faced such problem and as suggestion previously in our community, the people recommend for the pregnant mother to drink water and to chew onion to avoid any illness. This also true now days as we observed from a media (TV) they recommend to drink water and to use those herbal as they improve the health status of an individual. As such, previously we have not get any counseling from health professionals or concerned bodies regarding the causes for pre-term delivery. The other female participant stated that as we learned if any become pregnant it must avoid stress and crying high loaded material. If she stressed during pregnancy it result in disease or illness as it aggravated by pregnancy. During pregnancy, it possible to know the movement fetus in the womb by the mother at 5 and 6 month. Unit this month it difficult to now and the conceptus is more of water and as the fetus well formed during seven month. As such, care needed for pregnant mother even if the faced a stress as stress comes from different direction. As experience, foreigners give special care if one women become pregnant as if they provide flower when they get time to refresh her mind. Nevertheless, this not applied in our community and a women become married and having pregnancy, she faced different challenge and this may result for abortion.

One of the other FGD discussant (female) said that I have also a baby delivered in seven month and we challenged after delivery as a baby was unable to suck and we provided “Anchor” a processed powder milk. As I am very young and I have not now the pregnancy and I run while a knife in the neighbor cuts some. During that time, a gush of fluid pass and I waited for some period as thing for the causes and again the pass continue and I got to hospital. After assessment, they decided to do an operation after informed for me as the amniotic fluid completely drained. During that, the pregnancy was seven month. After that, the provided expressed breast milk for the baby by tube via nose until discharge from hospital, and we very challenged to grow until now as we provided “Anchor”. The other women stated the causes for pre-term delivery is that we go to health institution during 2-month pregnancy for checkup as health professionals informed to avoid carrying very loaded material until the fetus is mature. However, we have not avoided that think and we are struggled to stand up after carrying a material if no supporting individuals in nearby. As such, this create a great challenge in our community and causes complication on us. In addition, the other participant reported our community is also perception problem, if pregnant mother avoided or not willing to carry loaded material and other activity, the community rumor that does her pregnancy is unique, why she did not do that. Due to that case, the pregnant mother informed to do those activities. Our working farm field is very far from the living houses, we arrived at that area, and after working in the field and we re-back to home by carrying a banana, or mango, then even after arrived at home we further go to fetch water for cooking and for household utilizes. Again, we are also challenged to carry this water filled cans as it is loaded and this a causes for pre-term delivery. In addition, if the women faced fall accident, it also causes pre-term delivery.

One of the FGD participant (female) said that I provided pacifier for the baby as the mother already passed away due to the complication. I always ask myself or raise a question for me if the baby vomited the ingested material and feel abdominal discomfort. Consequently, I wash my hand, the pacifier and immerse it in hot water as I considered the hygiene problem my resulted for this condition. As the baby frequently vomit pass urine, and continuously managing such care giving care for pre-term baby is very challenging. Firstly, the baby continuously cry at the seventh month after delivery and I do not know why it cry. Nevertheless, the other individual has told to me that the baby cry to indicate a need for pacifier and provide for him as like a mother breast feed rather you only wrapped cannot subside his crying. As such, I provided a “muck” by adding to pacifier and the baby going to sleep after sucking. After that, I understand the situation as a baby need for breastfeed and this became clear for me. Until now, starting from seven month the baby feed by pacifier and even it does not try to suck my breast as a child who lucking the mother. The other participant also stated that, a pre-term baby exposed to sunlight after 15 days, as a mother warmth is not enough, but after 30days for term baby. The sunlight exposure very important for the baby to gain weight and the baby exposed the sunlight every other day. If the mother of the baby is available, the baby must get adequate breast milk until six month. I washed the body of my baby two times a day (in the morning and at night). During the time of washing and while I removed the cloth from the body, the baby exposed for cold and it becomes seriously ill, and we took to hospital. After admission, to the hospital oxygen provided via nasal cavity by tube, as my baby was faced difficulty of breathing. This condition occur during while the baby was at six month after delivery. I delivered at hospital and I only stayed for one day. B/c, the health care professionals tried to put the baby in the incubator. However, the baby continusely cry and unstable. My baby was very small and it only fillies a pist of my hand. I was very shay while I washing my baby as I only hold in one hand and it does not like a human being. I continuously pray to God and I have not wear a cloth rather after washing I putted in chest and hold it with a wrapped multiple cloths to make it hot until six month. Until six month, my baby does not going to sick and the health status was well. I continuously breast feed by putting in my chest. The baby cry if the body become very hot and I bathed to cool.

The other FGD participant stated I delivered my baby by operation and the baby was very small and I breastfeed by putting pillow to support. After discharging from hospital, the babies developed wound throughout the body and the baby semis like a “pen”. We challenged to manage and very stressed. My husband continuously pray to God for the condition why this happen in us. After that, the baby become well and the wound healed. When we took the baby to hospital, the health professional become angry to me that why this baby wasted as you are not the mother of the baby and you are well enough. Even if, I responded for them the baby delivered at seven month, but they cannot accepted my response. My baby breastfeed well, but they continuously vomit. We also bought artificial prepared milk or “Anchor” to provide for them, but the baby vomits. The other mother stated the seventh baby very small during birth as her bones easily observed the baby inhales and exhaled air, and it semis like a stick of moringa “shifaraw”. Even, I took a picture by my cellphone to show it while she grow. However, the sized increased and well in picture as compared the actual body. I provided a fresh butter at six month, and something like black worm structure was come out from the anus, and we were discussed with the my mother that this was cause that the baby becomes to be very small. B/c, there was the women who delivered at seventh with me and her baby semis like nine month baby. I have not put my baby in the bed, as the baby was very small even it was very difficult to differentiate the leg and the arm, and someone my sited on the baby. As such, the seventh baby needs critical care (care during breastfeeding, and washing the body) as a Doctor also informed. So, the mother very challenged, as he cannot cook food for him after a month like that of mother who delivered in nine month. I also very limited from different activities, and I stay like hen who hatch an egg.

Regarding challenges related to pre-term delivery, one of the participants stated that it is difficult to grow child as it needs close follow up, it limits from social interaction and other activities like going to market, attending social ceremonies, cooking food. The mother become hunger if some is not around her as the baby continuously cry and the mother is unable to cook food for him even after one month of delivery. The majority of the burden is similar to the mother who delivered at nine month. Nevertheless, it over weighs in the case of pre-term delivery. One of the mother reported that I had not attended in social grave or rituals, ceremonies and festivities for three years, and the neighbors, the kebele administrates also understand the situation and they supported me. The other FGD participant stated the breastfeed the child for two year or two year and half, and during this time the mother is challenged as she stay with baby for the whole day in our surrounding. However, educated mothers take rest while she going to work after breastfeeding and in our case there is not this opportunity and the mother has double burden (during pregnancy and delivery, and also during raring the child).

At last, the one of the FGD participant said that the health extension workers in our surrounding does not follow the mothers who delivered in seven month. They also not provide any information related to pre-term delivery rather than routine service like vaccination based on the ordered schedule and as provided in campaign. Those mothers are faced a great challenge and the peoples does not understand there situation due to lack of information on it. Furthermore, the other (male) discussant stated awareness creation by health professionals on pre-term delivery (on causes, if occur on caring aspects) needed in the community rather than others. Typically, knowledge creation needed for the mother and father who delivered pre-term baby. In addition, support from government via health professionals needed for the mother if her family is low income. As previously, the government provide “Aja” for those mothers who are low-income family. We can need money only from government rather counseling is very important for us. We thanks for your question and we are happy for providing this information for you as this information is important for you and for us. As you may report for concerned bodies and you are responsible to create awareness in public places as our community very negligent of information provided by HEWs (care providers in the surrounding). Our HEWs does now the information of the community. B/c, they does not update and frequently follow the community. As such, it is very important to use 1 to 5 members to create awareness and to get report directly as it needed by the government. In addition, counseling for the couples can be very important as most of the time husbands have no information and experience on caring baby and his wife faces most challenge. Women in the rural faced double burden as the cry different materials from far distance by her back. As such, education on those aspects needed to change the scenario. The other female discussant reported that the health professionals (HEWs) could not serve adequately and in companionate manner. If we go to health post, we cannot them, even in working hour as they close the office. They respond for us please do not disturb as I going to other activity, or meeting, and some of the time they open the office after 10:00AM. The kebele leaders know this situation, even if leaders come from woreda level and if they asked the community, the community does not forward the negative side of those professionals. I do not why the community lie on while leaders ask about the information of HEWs.

One of the IDI women defined preterm baby as born before nine month, or more commonly seven month. My baby was born in seven month and I do not know the birth weight. As my knowledge, there is no difference between preterm birth and low birth weight baby. She said the body of preterm babies are very soft and it seems the body white people. The cause preterm birth us carrying very loaded materials (carrying a sachet of potato and animal dung for fertilizer by the back), and doing other extra activities during pregnancy. My baby born due to carrying of high loaded material while I was in seven month pregnant. Nevertheless, the health extension workers also informed for all the pregnant mothers and with the husbands to avoid heavy loaded materials or equipment’s during pregnancy as it may harm the conceptus.

The care given for preterm babies are giving breast milk, and fresh cow milk and to prevent the child from germs from the milk, boiled alcohol is added to the fresh milk given by bottle. As fresh cow milk is important to strengthen the body of preterm babies and to grow faster. Health care provider recommend supplementary food for babies after six month. But, for the pre-term babies a “Muk” made from barley cereal is also given for the baby in addition to fresh cow mild. As recommendation from the women the fresh cow milk is not be boiled, if it is boiled the baby did not get the nutrients. To prevent from cold the baby always saddled by a cloth made from cotton.

The body of the preterm babies bathed after 6 days of delivery, and if the baby term it bathed after 4 days. During bathing, we put one hand on the cold to prevent from splash water and to make it dry. I bathed one side first only, and then shifted to the other. I faced a great challenge in caring my baby as it does not fully breastfeed and as most people said such like baby does not grow and it may die or pathway. As such, this creates stress on me. During bathing, it become syncope and in other time, again suddenly the body becomes cold. After breast feeding, feeding fresh com milk, it feel discomfort (the stool is like rounded rope), the stool is different to cases. As recommendation from my experience, it better if other additional supplements like fresh cow milk are avoided and the baby must bathed after 10 days expect after stooling the perineal areas is washed.

The support from the family and community are needed for the mother who faced such challenges like the husband and the mother-in-law, even neighbors must care for the baby and the mother must inform for those supports to avoid bathing. In addition, if there is any social problem the neighbors must inform for those family for here situation, if there is any “debo” activities again such family must excused, the neighbors bought any necessary supplies and sell on request of the mother from market. Unless, if there is no any supporting individual the mother must swaddle the baby with comfortable cloth before going for any activities. I faced different challenges while I keeping my baby as I unable to cook for me, limited social interaction, even I am unable to keep my personal hygiene.

The support from health workers are needed. The HP create awareness on preterm babies in house to house visit, how those babies carried, does it grow or not. I fell that my baby is lost due to my supplementation of fresh cow milk, due to lack knowledge on feeding preterm babies.

One of the IDI old male parent reported that one of the first male baby born at seven month and pathway. Again, the second baby was also born at seven month and survived until now. For me pre-term baby means a baby born before nine month. We counted the number of months after conception and the baby was born in seven month. We going to health care institution and they also informed two month is left to be the baby to mature. I do not the causes, as I have no information, but my wife breaking injera and the informed that “Mich” was one cause for pre-term birth for the first baby and this also true for the second baby even if she stopped the work before six month. Before five month we provided mother breast milk. Nevertheless, five month onward a “Muki” made from cereals was given for the baby. To prevent from cold I bought a cloth from market and we swaddling with that. In addition, to keep the baby hygiene we starting bathing after two of birth and bathed two three times period a day to make it comfortable. The baby was continuously crying and we challenged to manage, as we took to traditional healer in our surrounding as well as to health care institution. The neghoburs also said that this baby can’t survive as the first baby, and we are trouble with that information. After taking the medication (injection), the baby be calm. We also called my father and mother to apology as the cause may be “sin” or “Gome” from them. I also understated that, the “sin” cause the problem as my first and second baby born in seven month. To my understanding, the cause for pre-tern birth are heavy work, crying heavy load, baking injera during pregnancy. Regarding, the challenges, mostly my wife was reside in home to keep care for the baby and she is limited to do address other social issues. Nevertheless, I actively engaged in social activities like “Ikub”, “Idir”, “Yeho”, and “Debo”. The activity of my wife is severely, diminished as a baby is very weekend as compare to term babies, and it needs close follow up. As such, the neighbors now the situation, they give permission for her to keep her baby. My wife had follow up in the health center and she got counseling service from health care providers regarding how care for pre-term babies are given.

One of the IDI old traditional birth attendant said that I delivered a pre-term birth previously if the labor started on the way to health care institution and the baby is distressed. The pre-term are completely different from term babies as term babies are normal, but pre-term babies are very weak (the head is very little and the leg is also very weak). The families with income can provide good care for pre-term babies and the baby grow well, but others are challenged to keep. It is very difficult to bath as well as to feed pre-term babies. Some my through in the roadside by swaddling with a cloth. Most of the time pre-term babies are not survive and the families are stressed if they get pre-term baby.

One of the IDI old father of pre-term baby stated that we took my wife to health care institution due to severe pain. After assessment, the health care professionals reported that this is not a labor and they provided injection to subside the pain. Nevertheless, the baby delivered after two days of her visit, and admitted to NICU for 10 days. The baby was healthy, but the perception of peoples regarding pre-term babies are difficult, as baby at 8 month cannot survive. Because, my baby was born at 8 month after conception. Our community defined pre-term babies as babies born before nine month. Regarding, our baby it healthy, but the birth weight was very low and unable to feed, due that case it putted in incubator for ten days. After ten day, when the baby trained to breast feed, we contact with the mother. But, faced a challenge from the community that all are forwarded a comment that it is better if this baby was born in seven month as a baby born in eight month cannot survive. We also accepted such comment in some extent as we already live that community. I believed that 8-month baby is better than seven-month baby is, because the 8-month baby stayed a month long in the mother womb. Even, after discharge from hospital after eleven days, and come to home, most people believed that this baby might die after two or three days. Nevertheless, the baby survived until now without any problem. To may side, I haven’t now cause for pre-term birth. However, most people in our community stated they cause may a sin, “witchcraft” or “Ergiman”. The care given at home was as the baby skin was very weak as discharge due to case birth weight is more than 2k.g. We prevented a baby from a cold and a moving air by using a cloth made from cotton and we carefully wash the body as the skin cannot strengthen that much. Until, six month we only provided breast milk by giving special care for the mother. Previously, the skin was like a blood or the skin foreigner. We bathed every week to prevent from a cold while immersing frequently. After, passing stool we used a wipe to clean as washing with water or using other cloth are not much recommend for pre-term babies’.

We have not faced any challenge that much, except the community perception related with pre-tem babies. My personal life is not distorted due to the birth this child. Even, the community cannot believe the survival of this child until now. At the end, I suggested that the community perception must corrected as seven-month baby can survive but the eight month is not. This may be something like the community took as “Aliko”. I am evidence that 8 month baby can survive as it approached to term or nine month as compared with seven month baby.

One of the IDI old traditional birth attendant and traditional healer said pre-term baby means a baby delivered at seven month and if it delivered in nine month we call it has the baby is delivered in due date. We know pre-term birth, as we count the dates starting from the date of cessation of menses and if the baby delivered before nine month. I worked as traditional birth attendant for a long period, but I have not faced a pre-term baby.

We can differentiate pre-term from term baby, as pre-term babies are very weak and the body is soft. A term baby is strong and resist even if illness occur, but pre-term babies does not. The main cause for pre-term is fall accident, and crying very loaded materials. We can give care for pre-term babies by enclosing in cloth to avoid from cold, by good positioning in hand to warmth, and feeding by supplementing with nutritional foods. Frequent feeding to make it the baby to sleep and alternatively providing different food items. I have not provided a traditional medicine for pre-term babies until now. Nevertheless, provided for the other as the baby is continuously cry/shouting, unable to suck due to discomfort in abdomen, and unstable, and if does fail well after visiting health institution, I provided an herbal for the “evil eye”. I also massage the abdomen of the baby for “Bua”.
