# Supplementary material for: Community perceptions and experiences on caring for the premature babies in Arba Minch health and demographic surveillance site, southern Ethiopia: Interpretive Husserlian phenomenological study
Source: PLoS One. 2024 Jan 2;19(1):e0294155. doi: 10.1371/journal.pone.0294155 (PMC10760645; doi:10.1371/journal.pone.0294155)
Supplement: S1 File — (PDF) [file pone.0294155.s004.pdf]

Part I: Interview guide

A. Key informants background information

1. Key informant code:________
2. Age:_______
3. Sex: ______
4. Educational level: __________
5. Participant status:______

B. Interview script

1. What does it mean preterm baby for you? (**Probes:** would you explain further, difference with low birth weight babies)
2. How do you recognize preterm babies? (**Probes:** In what way, how we recognize, feature or characteristics)
3. What do you think about the causes of preterm birth? (**Probes:** maternal factors, general social factors, others)
4. What are the caring practices for preterm babies? (**Probes:** how we warmth, clarify regarding feeding, how we bath, when we bath, and state how we keep the hygiene of the preterm babies)
5. How do you experience in caring preterm or premature babies? (**Probes:** experiences during warmth, feeding, bathing, and hygiene)
6. What are challenges in caring for preterm babies? (**Probes:** as individual, as family, as community)
7. How those challenges affect the caring practices for preterm babies? (**Probes:** individual level challenges on caring practice, family and community level challenges as well)
8. Any additional points, you may state related to causes of preterm babies, recognition of preterm birth, practice and challenges in caring preterm babies. (**Probes:** participation of the community, availability of health care facility and support from health care providers to solve problems, maternity leave, transportation, income, etc.)

I completed the interview, thanks a lot!

Part II: Discussion guide

A. Participants background information

1. Participant code:________
2. FGD code: __________
3. Age:_______
4. Sex: ______
5. Educational level: __________
6. Participant status:______

B. FGDs script

1. What does it mean preterm baby for you? (**Probes:** would you explain further, difference with low birth weight babies)
2. How do you recognize preterm babies? (**Probes:** In what way, how we recognize, feature or characteristics)
3. What do you think about the causes of preterm birth? (**Probes:** maternal factors, general social factors, others)
4. What are the caring practices for preterm babies? (**Probes:** how we warmth, clarify regarding feeding, how we bath, when we bath, and state how we keep the hygiene of the preterm babies)
5. How do you experience in caring preterm or premature babies? (**Probes:** experiences during warmth, feeding, bathing, and hygiene)
6. What are challenges in caring for preterm babies? (**Probes:** as individual, as family, as community)
7. How those challenges affect the caring practices for preterm babies? (**Probes:** individual level challenges on caring practice, family and community level challenges as well)
8. Any additional points, you may state related to causes of preterm babies, recognition of preterm birth, practice and challenges in caring preterm babies. (**Probes:** participation of the community, availability of health care facility and support from health care providers to solve problems, maternity leave, transportation, income, etc.)

I completed the discussion, thanks a lot!
