# Supplementary figures and images for: Community perceptions and experiences on caring for the premature babies in Arba Minch health and demographic surveillance site, southern Ethiopia: Interpretive Husserlian phenomenological study
Source: PLoS One. 2024 Jan 2;19(1):e0294155. doi: 10.1371/journal.pone.0294155 (PMC10760645; doi:10.1371/journal.pone.0294155)

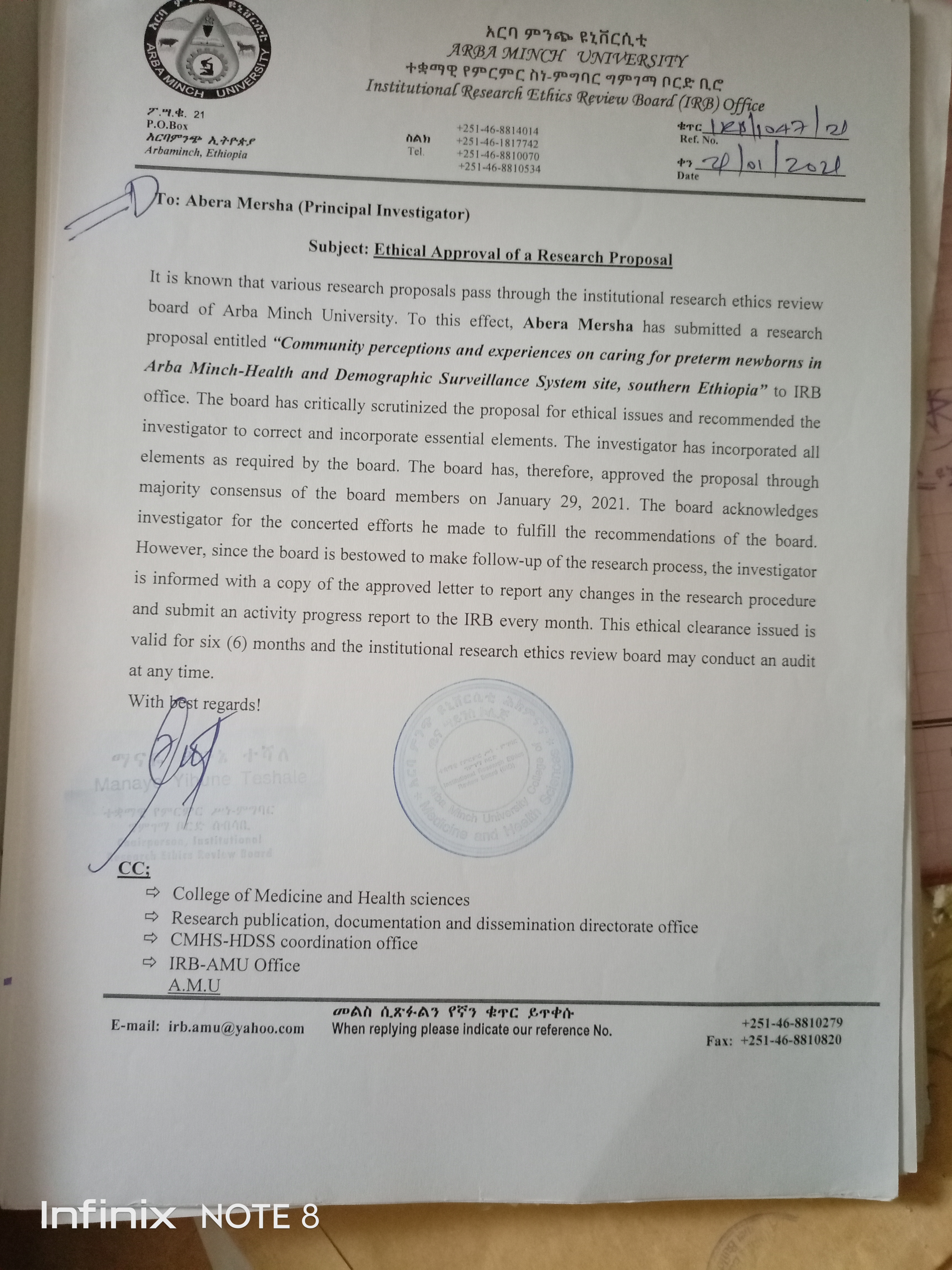

Supplement: S2 File — (JPG) [file pone.0294155.s005.jpg]
