# Supplementary material for: Community perceptions and experiences on caring for the premature babies in Arba Minch health and demographic surveillance site, southern Ethiopia: Interpretive Husserlian phenomenological study
Source: PLoS One. 2024 Jan 2;19(1):e0294155. doi: 10.1371/journal.pone.0294155 (PMC10760645; doi:10.1371/journal.pone.0294155)
Supplement: S1 Table — (DOCX) [file pone.0294155.s006.docx]

**Table 1: Developed main themes and sub-theme’s**

| **SNo** | **Main themes** | **Sub-themes** | ***Sub sub-theme*** |
| --- | --- | --- | --- |
|  | **General concept** | Definition |  |
|  |  | Difference with LBW |  |
|  | **Recognizing preterm babies** | Physical features | *Immature or the body is bloody* |
|  |  |  | *Very weak and small* |
|  |  | Limited range of motion due to neuromuscular immaturity | *Paralyzed*  *Does not flex and extend extremities* |
|  |  | Bizarre behaviors or characteristics | *Unstable and continuously cry* |
|  |  |  | *Unable to suck and breastfeed* |
|  | **Perceived causes for preterm birth** | Cause unknown |  |
|  |  | Maternal factor | *Being young* |
|  |  |  | *Caring high loaded materials* |
|  |  |  | *Fall accident* |
|  |  |  | *Health condition of the mother (hypertension, anemia, malaria, fever, stress, )* |
|  |  |  | *Family conflict* |
|  |  | Socio-cultural and spiritual factor | *“Mich”* |
|  |  |  | Sin (will of God) |
|  |  |  | *“Ergiman”* |
|  |  |  | Social influence to do extraneous activities |
|  |  |  | Witchcraft |
|  |  |  | *“Gome” or “Lanche”* |
|  |  |  | *“Evil eye”* |
|  | **Caring practices** | Warmth | *Put in cotton or cotton woolen* |
|  |  |  | *Skin to skin contact (kangaroo mother care (KMC)and kangaroo father care (KFC))* |
|  |  |  | *Sunlight exposure* |
|  |  |  | *Swaddling and wrapping with cloth* |
|  |  | Feeding | *Breastfeeding* |
|  |  |  | *Artificially prepared milk or formula feeding* |
|  |  |  | *Cow milk* |
|  |  |  | *Fresh butter* |
|  |  |  | *“Muk”* |
|  |  | Hygiene | *Frequent bathing* |
|  |  |  | *Washing and frequently changing clothes* |
|  |  |  | *Washing breast before feeding* |
|  |  |  | *Keep the hygiene of the equipment’s used for the baby* |
|  |  |  | *Use wipe to clean after passing stool* |
|  |  | Limit visiting |  |
|  |  | Physical protection |  |
|  | **Support for the mother with preterm baby** | Community or social support |  |
|  |  | Family support |  |
|  |  | Health professional’s support |  |
|  |  | Government support |  |
|  | **Challenges** | Difficult to feed |  |
|  |  | Difficult to bath |  |
|  |  | Limit social participation |  |
|  |  | Prone for infection or any disease |  |
|  |  | Psychosocial and economic impact |  |
|  |  | Lack of support from husband |  |
|  | **Compulsory action** | Lack of hospital in the surrounding |  |
|  |  | Lack of support from local health workers (HEWs) |  |
|  |  | Poor road construction and lack of transportation |  |
