# Supplementary material for: Community perceptions and experiences on caring for the premature babies in Arba Minch health and demographic surveillance site, southern Ethiopia: Interpretive Husserlian phenomenological study
Source: PLoS One. 2024 Jan 2;19(1):e0294155. doi: 10.1371/journal.pone.0294155 (PMC10760645; doi:10.1371/journal.pone.0294155)
Supplement: Attachment — Submitted filename: Response to Reviewers.docx [file pone.0294155.s007.docx]

**Response to Reviewers**

**Manuscript Number:** PONE-D-23-19601

**Manuscript Title:** Community perceptions and experiences on caring for the premature babies in Arba Minch Health and Demographic Surveillance Site, southern Ethiopia: Interpretive Husserlian phenomenological study

**Dear Editor,**

Thank you for your time and constructive feedback on our manuscript. I have responded to each reviewer's comments and suggestions in detail below.

| **Comments** | **Response** |
| --- | --- |
| **Academic Editor** | |
| **Journal requirements:** | |
| Please ensure that your manuscript meets PLOS ONE's style requirements, including those for file naming | I thank you for giving this suggestion to cross-check the journal requirements. I cross-checked the PLOS ONE's style requirements and the manuscript meets the requirements. |
| We note that the grant information you provided in the ‘Funding Information’ and ‘Financial Disclosure’ sections do not match. | Those information directly submitted or inputted to the system rather than included in the manuscript. It was checked and the information included was correct, b/c Arba Minch University provided a minimal fund by the stated grant number but had no role in study design, data collection and analysis, decision to publish, or preparation of the manuscript. |
| In your Data Availability statement, you have not specified where the minimal data set underlying the results described in your manuscript can be found. | Those information directly submitted or inputted to the system rather than included in the manuscript. The minimal data set underlying the results was described in the revised manuscript (included as supporting information). |
| Please review your reference list to ensure that it is complete and correct. | I cross-checked and all the references were complete and correctly stated. |
| **Reviewer #2** | |
| -Grammatical error should be corrected  - The English language needs to be improved in the text.  - Proofreading is required | The grammatical errors were corrected, the English language was improved, and the manuscript was proofread. |

***Best Regards!***

***Shitaye Shibiru,***

***On behalf of co-authors***
